# Interplay between angiopoietin-2, vascular endothelial growth factor and peroxynitrite is an important determinant of vascular hyperpermeability during methicillin-resistant *Staphylococcus aureus *sepsis

**DOI:** 10.1186/cc10408

**Published:** 2011-10-27

**Authors:** P Enkhbaatar, Y Zhu, L Traber, D Traber

**Affiliations:** 1 Department of Anesthesiology, University of Texas Medical Branch, Galveston, TX, USA

## Introduction

We have reported that nitric oxide (NO) production and microvascular hyperpermeability were significantly higher in septic sheep with methicillin-resistant *Staphylococcus aureus *(MRSA) than with *Pseudomonas aeruginosa*. We hypothesize that peroxynitrite, a byproduct of NO, causes vascular hyperpermeability in MRSA sepsis via promoting vascular endothelial growth factor (VEGF) and angiopoietin-2 (Ang-2). The hypothesis was tested, using both a well-established ovine sepsis model and cultured human umbilical endothelial cells (HUVECs).

## Methods

Female ewes were chronically instrumented with multiple catheters and live MRSA (USA300, 10^11 ^CFU) was instilled into the both lungs by bronchoscope under deep isoflurane anesthesia. The sheep were then randomly allocated to control and treated (nonspecific NOS inhibitor L-NAME, 25 mg/kg, i.v., every 12 hours) groups and monitored for 24 hours for cardiopulmonary hemodynamics. The cells were challenged with 10^5 ^CFU of live MRSA or 50 μM peroxynitrite and co-incubated with or without L-NAME, peroxynitrite scavenger FeTMPyP, Tie-2 and Ang-2 antibody, and VEGF and its antibody. At different times after the treatment, the permeability was measured by quantifying the amount of FITC-Dextran that passed through the confluent HUVEC monolayer (*n *= 4). Ang-2 mRNA was determined by RT-PCR in those cells with or without treatment as well (*n *= 4). Statistical analysis: one-way ANOVA (Bonferroni).

## Results

*In vivo*, L-NAME significantly reduced MRSA-induced fluid accumulation and requirement, as well as expression of VEGF. HUVEC permeability was time-dependently increased following MRSA co-incubation, reaching a plateau at 2 and 4 hours. These permeability changes (73 ± 4 RFUs) were significantly (*P *< 0.001) inhibited by 1 mM L-NAME (28 ± 1), 5 μM FeTMPy (34 ± 2), 5 μg/ml Tie-2 antibody (32 ± 2), and 5 μg/ml Ang-2 antibody (30 ± 1). In HUVECs, the Ang-2 mRNA was time-dependently (picks at 30 minutes) and dose-dependently increased by peroxynitrite (highest at 50 μM). Treatment of HUVECs with 5 μM VEGF augmented the MRSA-induced Ang-2 mRNA increases. The latter was reversed with FeTMPyP and 5 μM VEGF antibody.

## Conclusion

Ang-2 and VEGF, Tie-2 receptor, NO and its byproduct peroxynitrite play an important role in MRSA-induced vascular hyper-permeability. The results strongly suggest that peroxynitrite increases vascular hyper-permeability by promoting Ang-2 release through stimulating the VEGF expression during MRSA-induced Gram-positive sepsis.

